# A Pilot Study of Infrared Thermography Based Assessment of Local Skin Temperature Response in Overweight and Lean Women during Oral Glucose Tolerance Test

**DOI:** 10.3390/jcm8020260

**Published:** 2019-02-19

**Authors:** Bushra Jalil, Valentina Hartwig, Davide Moroni, Ovidio Salvetti, Antonio Benassi, Zunera Jalil, Laura Pistoia, Tommaso Minutoli Tegrimi, Alfredo Quinones-Galvan, Giorgio Iervasi, Antonio L’Abbate, Letizia Guiducci

**Affiliations:** 1 Istituto di Scienza e Tecnologie dell’Informazione “Alessandro Faedo” CNR, 56124 Pisa, Italy; davide.moroni@isti.cnr.it (D.M.); ovidio.salvetti@isti.cnr.it (O.S.); antonio.benassi@isti.cnr.it (A.B.); 2 Istituto di Fisiologia Clinica CNR, 56124 Pisa, Italy; iervasi@ifc.cnr.it (G.I.); segrlabb@ifc.cnr.it (A.L.); letiziag@ifc.cnr.it (L.G.); 3 Department of Computer Science, Air University, Islamabad 44000, Pakistan; zunera.jalil@mail.au.edu.pk; 4 Fondazione G. Monasterio CNR-Regione Toscana, 56124 Pisa, Italy; laura.pistoia@ftgm.it (L.P.); tommaso.minutoli@gmail.com (T.M.T.); quinones@ftgm.it (A.Q.-G.); 5 Scuola Superiore Sant’Anna, Institute of Life Sciences, 56127 Pisa, Italy

**Keywords:** obesity in females, infrared thermography, regional skin temperature, oral glucose tolerance test (OGTT)

## Abstract

Obesity is recognized as a major public health issue, as it is linked to the increased risk of severe pathological conditions. The aim of this pilot study is to evaluate the relations between adiposity (and biophysical characteristics) and temperature profiles under thermoneutral conditions in normal and overweight females, investigating the potential role of heat production/dissipation alteration in obesity. We used Infrared Thermography (IRT) to evaluate the thermogenic response to a metabolic stimulus performed with an oral glucose tolerance test (OGTT). Thermographic images of the right hand and of the central abdomen (regions of interests) were obtained basally and during the oral glucose tolerance test (3 h OGTT with the ingestion of 75 g of oral glucose) in normal and overweight females. Regional temperature vs BMI, % of body fat and abdominal skinfold were statistically compared between two groups. The study showed that mean abdominal temperature was significantly greater in lean than overweight participants (34.11 ± 0.70 °C compared with 32.92 ± 1.24 °C, *p* < 0.05). Mean hand temperature was significantly greater in overweight than lean subjects (31.87 ± 3.06 °C compared with 28.22 ± 3.11 °C, *p* < 0.05). We observed differences in temperature profiles during OGTT between lean and overweight subjects: The overweight individuals depict a flat response as compared to the physiological rise observed in lean individuals. This observed difference in thermal pattern suggests an energy rate imbalance towards nutrients storage of the overweight subjects.

## 1. Introduction

Obesity is recognized as a major public health issue, as it is linked to the increased risk of severe pathological conditions such as arterial hypertension, diabetes, coronary heart disease, and many others [1]. These negative effects of obesity have attracted many researchers to explore its effects on human health. Infrared Thermography (IRT) is a well-known technique which detects infrared energy emitted from an object, converts it to temperature, and displays the image of temperature distribution [2]. In the past few decades, IRT has been used to study diseases in which changes in skin temperature could indicate inflammation or blood flow alterations, measuring skin surface temperature [3]. The advantage of using this technique, compared to alternative methods requiring contact between the body surface and the sensor, lies in the fact that with the use of IRT the skin temperature is not influenced by the presence of any probe that could modify the temperature through conduction or irradiation. In that sense, the use of IRT in the measurement of human skin temperature has the advantage of being completely noninvasive and to record temperature data simultaneously from different points on a wide area of the body [3]. Savastano et al. [4] have studied human obesity in relation to the abdominal and right hand temperature variation using such cameras. They examined obese adults (both males and females) as compared to normal weight adults. Analysis of data indicated that obese participants demonstrated significantly lower abdominal temperature patterns than those of their normal weight counterparts. Chudecka et al. [5] have drawn a temperature map of obese women and highlighted the body areas where heat transfer is particularly impeded. The above study attempted to examine the potential relationship between body composition (including subcutaneous and visceral fat distribution) determined by bioimpedance as well as BMI (body mass index), and skin surface temperature distribution recorded at rest.

Recently, it has been demonstrated that brown adipose tissue (BAT) is a key regulator in energy balance and a major contributor to diet-induced thermogenesis (DIT) also in adults [6]. This fact motivated the research of new methodologies for the detection of BAT in a non-invasive way. Many authors have recently demonstrated that thanks to thermal imaging, the exact contribution of specific environmental, dietary, and lifestyle interventions on brown adipose tissue thermogenesis can now be quantified. Our group [7] designed a noninvasive multimodal imaging technique to identify and monitor temperature variation related to human BAT in adults with different BMI. The preliminary results of that study showed that near-infrared spectroscopy (NIRS) together with IRT in the supraclavicular area might be a novel method for monitoring, during standard clinical practice, the diet intervention which aims to stimulate BAT as a potential therapeutic target against obesity and diabetes.

Starting from the well-established fact that the resting metabolic heat production is significantly greater in obese than in lean individuals [8], our hypothesis in this pilot study is to find a difference in temperature profiles between lean and overweight subjects in response to diet-induced thermogenesis.

We chose the abdomen and hand as body areas to be investigated since we were not interested in detecting BAT activation but in evaluating the relations between adiposity (and biophysical characteristics) and temperature profiles under thermoneutral conditions.

Regarding the choice of stimulus to induce thermogenesis, we chose to use an oral glucose tolerance test (OGTT) to be able to standardize the thermogenic stimulus induced by the meal as accurately as possible and in a highly reproducible way among experiments [9,10]. In this way, it was possible to analyze temperature variations without using another type of stimulus that could have caused vasoconstriction, such as the cold stimulus that would have affected the thermal response observation [4,11].

To our knowledge, this is the first study that uses IRT to evaluate differences in skin temperature of different body areas between lean and overweight subjects at rest condition and during a caloric load administration.

## 2. Methods

### 2.1. Infrared Thermography

An infrared camera is a camera system endowed with a sensor that detects infrared energy from an object and uses this information to estimate pointwise the temperature producing a thermographic image of the object itself. When imaging the skin, infrared cameras are capable of inferring skin temperature on the basis of radiated energy. It is important to notice that radiated energy does not account for the totality of heat transferred from the body to the environment, since other processes such as conduction and, more notably, convection should be taken into account [12]. Evaporation also contributes to heat dissipation. This study is limited to the evaluation of skin temperature, while quantification of the total transferred heat is beyond the current scope.

Thermographic images of the hand and abdomen were acquired by using a Fluke Ti9Thermal Imagers (Fluke Corporation, Everett, WA, USA). The camera works well for surfaces that are efficient at radiating energy (high emissivity), such as the skin that has an emissivity factor of 0.98 [3,13]. Moreover, the infrared thermography camera has a 160 × 120 mm focal plane array; it operates in the 7.5 μm to 14 μm wavelength range, with 5% accuracy and a thermal sensitivity (NETD) ≤0.2 °C at 30 °C (200 mK). The camera works in the range of −20 °C to +250 °C and auto calibrates itself if above −10 °C as explained in the technical specifications provided by the manufacturer [14]; this allows estimation of the absolute temperature of body surface, without having the necessity to manually perform calibration with a reference object at each acquisition session. Thermographic images of abdomen and right dorsal hand were acquired for each subject at different time steps (see Section 2.3); at each time step, the subject was positioned in the same position by means of references placed on the examination table and with a marker drawn directly on the area of interest. The marker was also used to choose the region of interest in the processing phase. In order to assure that the IRT camera was placed at the same distance from the subject every time, we used a reference on the area of interest to adjust the automatic focus of the camera.

### 2.2. Subjects

Five healthy women (age 31.2 ± 8.3) with low body mass index (BMI = 19.8 ± 2.4 kg/m^2^, body fat = 25.2 ± 4.6%) and five overweight healthy women (age 38.9 ± 14.0) (BMI = 28.6 ± 3.1 kg/m^2^, body fat = 40.2 ± 2.2%) were studied. Table 1 shows the characteristics of all subjects. The study was authorized by the local Ethical Committee. Informed consent was obtained from all individual participants included in the study. Subject 1 had anthropometric characteristics (abdominal skin fold and body fat percentage) that differ to the other subjects in the lean group due to the android distribution of the subcutaneous abdominal fat mass. However, the chosen classification criteria to distinguish the two groups was BMI: A subject with BMI value < 24.9 was classified in the lean group, while a subject with BMI value ≥ 24.9 was classified in the overweight group.

### 2.3. Experimental Design and Protocols

All subjects were selected from the metabolic unit of Fondazione G. Monasterio CNR—Regione Toscana, Pisa, Italy. Firstly, height and weight were measured and then BMI was calculated. Abdominal skin fold was also measured in all subjects using a caliper (Skinfold Caliper Gima Mod 27320 [15]).

Moreover, thermographic baseline images were collected (after a 15-min acclimatization period). A 3-h oral glucose tolerance test with the ingestion of 75 g of oral glucose was performed. IRT images were taken every hour for 180 min. The 2 h OGTT is generally used for glucose tolerance/diabetes diagnosis. Instead, to study the glucose metabolism, in particular the rate of glucose disappearance, the time requested is calculated from the relationship with the standardized method (clamp test) and it is 3 h [16]. The ambient temperature was controlled and remained constant (20 °C) for all measurements. All the measurements were performed in the spring. Regional skin temperature (°C) values were extracted from the region of interest (ROI) in hand and abdomen and correlated with % of body fat. We choose to use an OGTT as stimulus for two reasons: first, the OGTT is routinely used in standard clinical practice, so in this way there is no need to use a further stimulus for the analysis of thermal response; second, OGTT does not cause vasoconstriction as is the case for cold exposure stimuli, which could affect the thermal response observation [11].

### 2.4. Statistical Analysis

Intragroup differences of temperature over time were evaluated by Student’s t-test for paired samples (variables were tested for normality using the Shapiro-Wilk normality test), while the differences in parameters between the two groups were evaluated by ANOVA. A *p* value < 0.05 was considered to be significant. Correlation tests have been performed using Pearson correlation. All analyses were conducted using MATLAB 2014a.

## 3. Results

**Figure 1:**
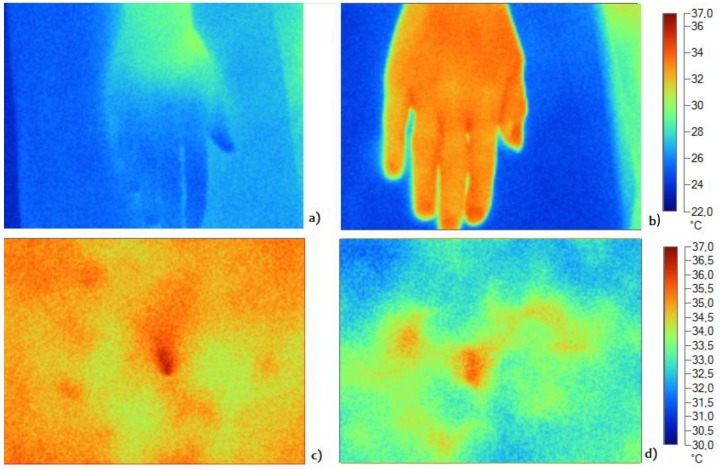
Example of an IRT image of hand and abdomen of a lean (**a**,**c**) and an overweight (**b**,**d**) subject at rest. Hand temperature is higher in the overweight subject compared to the lean subject, while abdominal temperature is higher in the lean subject.

By way of an example, Figure 1 shows the temperature image in the baseline condition for the hand (Figure 1a,b) and the abdomen (Figure 1c,d) of a lean subject (Figure 1a,c) and an overweight subject (Figure 1b,d). Color scale at the right side of the picture indicates the temperature values (°C).

**Figure 2:**
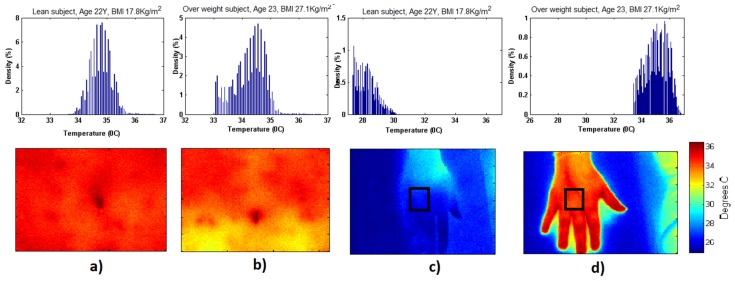
Thermal images of hand and abdomen of two subjects with matching age but different BMI, (**a**) lean subject abdomen: Gaussian-like thermal distribution, (**b**) overweight subject abdomen: non-Gaussian thermal distribution; (**c**) Lean subject hand: non-Gaussian thermal distribution, (**d**) Overweight subject hand: non-Gaussian thermal distribution.

The density graph of two subjects belonging to the same age group (22 ± 1 year) but with different BMIs (22 years with 17.8 kg/m^2^ and 23 years with 27.1 kg/m^2^) are shown in Figure 2. We observed a normal Gaussian-like thermal distribution on the abdominal area in lean subjects compared to the overweight subjects, where we observed irregular thermal distribution and relatively lower temperature distribution. On the contrary, we observed non-Gaussian thermal distribution on the hands of both lean and overweight subjects. However, we observed lower temperature density in the case of the lean subjects and relatively higher temperature density in the overweight subjects, indicating that overweight subjects had increased heat dissipation through the hands. Based on our findings, we believe that the temperature distribution of these data predicts the spectral distribution of two groups. However, with this limited data it is difficult to draw a conclusion with full confidence regarding Gaussian/non-Gaussian behavior of two groups.

**Figure 3:**
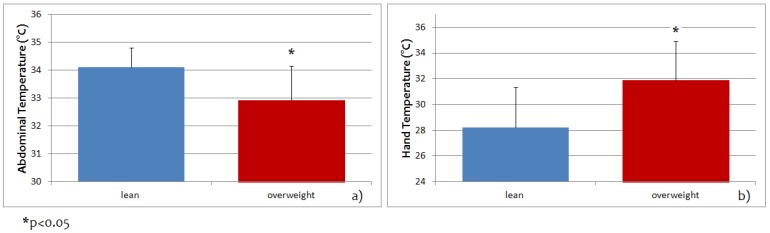
Abdominal (**a**) and hand (**b**) temperature at the baseline for lean and overweight groups. Statistically significant differences between the two groups are found both in abdominal and hand temperature, with the opposite trend.

Figure 3 shows abdominal and hand temperature at the baseline for the lean and overweight groups.

Mean abdominal temperature was significantly greater in lean than overweight participants (34.11 ± 0.70 °C compared with 32.92 ± 1.24 °C, *p* < 0.05, Figure 3a). Mean hand temperature was significantly greater in overweight than lean subjects (31.87 ± 3.06 °C compared with 28.22 ± 3.11 °C, *p* < 0.05, Figure 3b).

**Figure 4:**
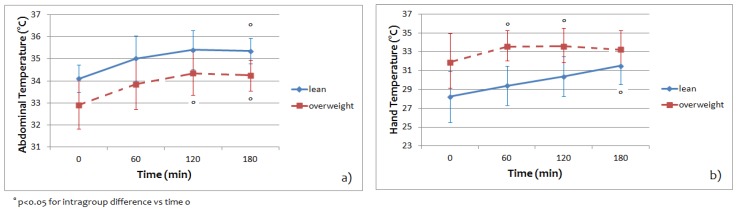
Abdominal (**a**) and hand (**b**) temperature values averaged on five lean subjects and on five overweight subjects, together with the 95% confidence intervals, during oral glucose tolerance test (OGTT).

Figure 4 shows the regional temperature averaged on five lean subjects and on five overweight subjects for abdomen and hand area during the entire experimental paradigm.

At the baseline and during the entire OGTT, the abdominal temperature of the lean group was higher respect to the overweight group. For the hand temperature, instead, the overweight group had a relatively higher temperature with respect to the lean group at the baseline and also during the entire OGTT.

**Figure 5:**
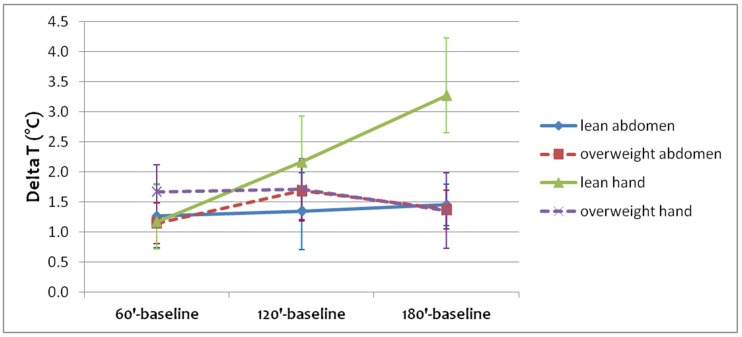
Temperature changes during OGTT with respect to rest (baseline, that is Time 0′), together with the 95% confidence intervals. For the lean group, both abdominal and hand temperature continues to increase, while for the overweight group abdominal and hand temperature increases only during the first two hours of OGTT.

Figure 5 shows the differences between mean temperature during the OGTT time steps (60′, 120′, and 180′) and the mean temperature at rest (baseline, that is Time 0′) in abdominal and hand regions for lean and overweight subjects, together with the 95% confidence intervals. While for the lean group the temperature of both areas continues to increase, for the overweight group the temperature of abdominal and hand areas increases more during the first two hours of OGTT.

**Figure 6:**
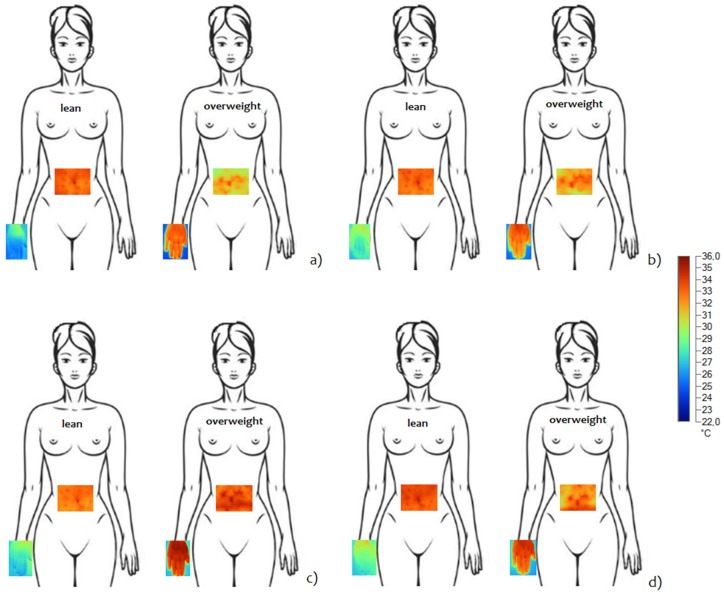
Abdominal and hand patterns of thermal response during OGTT for a lean subject and an overweight subject: (**a**) baseline condition, (**b**) 60′ after glucose ingestion, (**c**) 120′ after glucose ingestion, (**d**) 180′ after glucose ingestion.

Figure 6 shows the thermal images acquired during OGTT in a lean subject (left side) and an overweight subject (right side), both for the abdomen and hand. In this example, the differences between the two thermal patterns are well observable for both areas of measurement.

**Figure 7:**
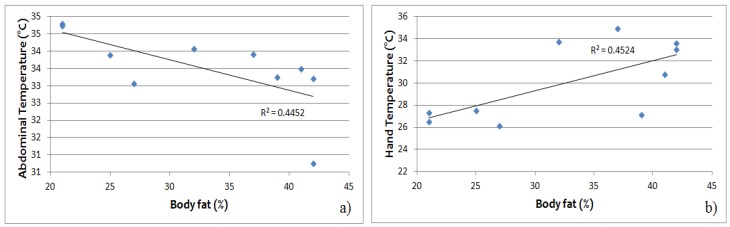
Baseline (**a**) abdominal and (**b**) hand temperature relative to the percentage of total body mass from fat in lean and overweight participants. The abdominal temperature is negatively related to body fat (*R*^2^ = 0.445, *p* < 0.05), while hand temperature is positively related to body fat (*R*^2^ = 0.452, *p* < 0.05).

In a regression model relating baseline temperatures measured by IRT to body fat (%), the abdominal temperature was negatively related to body fat (*R*^2^ = 0.445, *p* < 0.05) (Figure 7a), while hand temperature was positively related to body fat (*R*^2^ = 0.452, *p* < 0.05) (Figure 7b).

## 4. Discussion

Thermal imaging has been used to observe the relationship between morphology, body composition, and obesity [5,17,18], and also to examine heat production and dissipation in obese adults as compared to normal weight adults [4]. Moreover, the use of IRT is being increasingly recognized as a valid and complementary method to standard imaging modalities (PET/CT) for the detection of BAT activation as a potential treatment target for metabolic conditions [19,20,21,22]. All the studies relative to the use of IRT for the BAT activation detection generally employ a cold exposure (i.e., hands and/or feet immersion in ice-cold water or cold water pumped through a cooling vest): this kind of test is not easily usable during clinical practice. Since the differences in body surface temperature between lean and overweight subjects at rest or in different environmental conditions have been already demonstrated, our hypothesis was that IRT could be used during a more suitable test to achieve information regarding the use of body surface temperature as an indicator in the pathogenesis of obesity. The main aim of the study was to analyze temperature variations in normal and overweight subjects by evaluating the thermal images of the hand and of the abdomen under thermoneutral conditions and during an OGTT, as routinely used in standard clinical practice. We observed a normal Gaussian-like thermal distribution on the abdominal area in lean subjects as compared to the overweight subjects (Figure 2), where the distribution was more irregular with relatively lower temperature values. On the contrary, we observed non-Gaussian thermal distribution on the hands of both lean and overweight subjects. These results demonstrate the utility of IRT method able to show local heterogeneity in thermal distribution, allowing the placement of dermal thermistors on warm or cool areas of the skin more reliably than by depending on standard torso sites or other methods [18,23].

Regarding the comparison of thermal response in lean and overweight subjects, we observed that overweight participants, as compared to the lean subjects, had lower abdominal skin temperature than in the hand indicating relatively higher temperature in overweight subjects. This obtained lower abdominal skin temperature is in agreement with the literature, and indicates that a high body fat percentage is associated with low skin surface temperature [4,24,25]. The phenomenon was originally observed in 1937 [26] using a method of partitional calorimetry.

In fact, DIT comprises two parts, an obligatory component of energy required for the processing, transport, and storage of nutrients from the meal and a facultative component of heat energy [27]. An interesting finding of the present study is the differences in temperature profiles during OGTT between lean and overweight subjects: the overweight individuals depict flat responses as compared to the physiological rise observed in lean individuals. This difference observed corresponds to a facultative component of heat energy. Therefore, considering that the post-prandial energy expenditure represents the sum of heat energy that is lost and chemical energy that is stored after a meal, the differences observed in thermal patterns between normal and overweight individuals suggest possible shifts in energy balance that warrant further investigation [27].

We detected, in both groups, a consistent and highly localized increase in local temperature of hand and abdomen areas induced by glucose ingestion (Figure 4). It is even more interesting to note that both the abdominal and hand temperature in lean subjects continued to increase during the OGTT. Abdomen temperature in overweight subjects changed more during the first 2 h of OGTT protocol with respect to the temperature of the same area in lean subjects, while the hand temperature remained more or less constant during the entire experimental protocol (Figure 5). Our pilot study relative to the thermal pattern in the abdomen and hand regions (Figure 6) suggests that larger variations in skin temperature distributions occur as body fat increases [28]. These variations are present both at rest and during the entire experimental protocol, to indicate a different thermal response between the two groups. Our analysis shows differences not only in the mean temperature of the chosen areas but also in the distribution of the temperature, a fact that is not possible to observe with single point measurements. Differences between the lean and overweight subjects have been verified in our study by the correlations between the body fat percentage and the measured skin temperatures (Figure 7).

The positive relation between hand temperatures and body fat percentage underlines the relative importance of distal body sites for heat dissipation in overweight subjects.

One limitation of this study is the limited size of the sample that prevents generalized results: In fact, the observed non-Gaussian distributions in some subjects could be due to individual variability in thermogenesis and heat patterning. However, in light of the results obtained, the described study design can be easily applied to future studies including a larger sample size.

Finally, it must be noted that, in this study, the response of participants was evaluated by means of temperature profiles, without referring to the actual heat transferred by each region of interest. In the future, it would be of interest to analyze the effect of caloric load administration on different subjects with respect to regional heat losses. To this end, a precise heat transfer model can be introduced, possibly taking into account convection and evaporation.

## 5. Conclusions

In this paper, we have applied non-invasive infrared imaging technique in order to monitor temperature variations in lean and overweight females with different body mass indices during OGTT. We observed different thermal patterns in abdominal and hand regions of lean as compared to the overweight subject.

Despite being a pilot study, this work represents a good starting point to validate our findings further on a larger data set in different physical conditions and with obesity-related diseases. The final clinical protocol could include IRT investigations of further body areas (such as neck and supraclavicular area) together with other non-invasive methodologies such as NIRS [7], in order to study diet-induced thermogenesis in obesity.

## Figures and Tables

**Table 1:** Participant characteristics.

		Sex	Age (years)	Weight (Kg)	BMI (Kg/m^2^)	Abdominal Skin Fold (mm)	Body Fat (%)
**Lean**	Subject 1	F	31	62.7	23.6	32.5	32
	Subject 2	F	33	53.0	19.0	17.0	27
	Subject 3	F	22	50.4	17.9	8.0	21
	Subject 4	F	44	41.5	18.0	6.0	21
	Subject 5	F	26	51.0	20.4	10.0	25
**Over weight**	Subject 6	F	55	73.0	28.2	24.0	42
	Subject 7	F	48	67.0	24.9	27.0	41
	Subject 8	F	43	97.7	33.4	32.5	42
	Subject 9	F	23	67.0	27.2	32.5	37
	Subject 10	F	26	91.0	29.2	36.0	39
